# Use of 1-MNA to Improve Exercise Tolerance and Fatigue in Patients after COVID-19

**DOI:** 10.3390/nu14153004

**Published:** 2022-07-22

**Authors:** Michał Chudzik, Monika Burzyńska, Joanna Kapusta

**Affiliations:** 1 Medical Center, Saint Family Hospital, 90-302 Lodz, Poland; michalchudzik@wp.pl; 2 Department of Epidemiology and Biostatistics, Social and Preventive Medicine of the Medical University of Lodz, 90-752 Lodz, Poland; monika.burzynska@umed.lodz.pl; 3 Department of Internal Medicine and Cardiac Rehabilitation, Medical University of Lodz, 70-445 Lodz, Poland

**Keywords:** COVID-19, MNA, chronic fatigue syndrome, post-COVID syndrome

## Abstract

COVID-19 is not only a short-term infection, as patients (pts) recovering from SARS-CoV-2 infection complain of persisting symptoms, which may lead to chronic fatigue syndrome. There is currently no evidence that nutritional supplements can assist in the recovery of pts with chronic fatigue syndrome. 1-Methylnicotinamide (1-MNA) is an endogenic substance that is produced in the liver when nicotinic acid is metabolized. 1-MNA demonstrates anti-inflammatory and anti-thrombotic properties. Therefore, we investigated whether 1-MNA supplements could improve exercise tolerance and decrease fatigue among patients recovering from SARS-CoV-2. Methods: The study population was composed of 50 pts who had recovered from symptomatic COVID-19. The selected pts were randomized into two groups: Gr 1 (NO-1-MNA)—without supplementation; Gr 2 (1-MNA) with 1-MNA supplementation. At the beginning of the study (Phase 0), in both groups, a 6-minute walk test (6MWT) was carried out and fatigue assessment was performed using the Fatigue Severity Scale (FSS). Both FSS and 6MWT were repeated after 1 month. Results: A significant improvement in the mean distance covered in the 6MWT was noted at follow-up in Gr 1-MNA, compared with Gr NO-1-MNA. We also noted that in Gr 1-MNA, the 6MWT distance was significantly higher after 1 month of supplementation with 1-MNA, compared with the beginning of the study (515.18 m in Phase 0 vs. 557.8 m in Phase 1; *p* = 0.000034). In Gr 1-MNA, significantly more pts improved their distance in the 6MWT (23 out of 25 pts, equal to 92%), by a mean of 47 m, compared with Gr NO-1-MNA (15 of 25 pts, equal to 60%) (*p* = 0.0061). After one month, significantly more patients in the group without 1-MNA had severe fatigue (FSS ≥ 4) compared with the group with supplementation (Gr 1-MNA = 5 pts (20%) vs. Gr NO-1-MNA = 14pts (56%); *p* = 0.008). Conclusions: 1-MNA supplementation significantly improved physical performance in a 6-min walk test and reduced the percentage of patients with severe fatigue after COVID-19. The comprehensive action of 1-MNA, including anti-inflammatory and anticoagulant effects, may be beneficial for the recovery of patients with persistent symptoms of fatigue and low tolerance to exercise after COVID-19.

## 1. Introduction

Coronavirus disease 2019 (COVID-19) is a serious respiratory disease that results from infection with a newly discovered coronavirus (SARS-CoV-2). The number of COVID-19 survivors is increasing daily. Unfortunately, COVID-19 is not only a short-term infection. Many studies have revealed that patients (pts) recovering from SARS-CoV-2 infection complain of persisting symptoms [1] including fatigue, diffuse myalgia and weakness, which may lead to chronic fatigue syndrome [2,3]. Fatigue and limited exercise tolerance are some of the most common symptoms, reported by 34% to 69% of COVID-19 patients [4,5,6,7]. Many clinical trials have investigated ways to manage these post-COVID symptoms. Unfortunately, no specific treatment for post-COVID-19 fatigue has yet been introduced into clinical practice [8].

Chronic fatigue syndrome (CFS) is characterized by severe and disabling fatigue without a patho-physiologic explanation [8]. According to A.S. Bansal et al. [9] persistent fatigue lasting more than 6 months may be observed in several viral and bacterial infections. Many studies of post-viral fatigue and CFS focus on immune dysregulation and activation caused by changes in cytokine levels [10,11,12]. These changes can lead to microvascular thrombosis and endothelial dysfunction with impaired microcirculatory function, which may explain some post-COVID-19 related symptoms and complications [13,14]. It is likely that multiple and/or synergistic causal mechanisms underlie post-COVID-19 syndrome.

There is currently no evidence that nutritional supplements can assist in the recovery of pts with chronic fatigue syndrome. Nonetheless, given that the potential causes of the main symptoms of post-COVID-19 (i.e., limited exercise tolerance and fatigue) include inflammation, endothelial damage, microcirculatory thrombosis and metabolic disorders leading to insufficient production of energy, we should look for supplements that act comprehensively on all these pathogenetic mechanisms. One such molecule is 1-Methylnicotinamide (1-MNA), which is the methylated amide of nicotinic acid (niacin, vitamin B3). 1-Methylnicotinamide is an endogenic substance that is produced in the liver when nicotinic acid is metabolized. 1-MNA demonstrates anti-inflammatory and anti-thrombotic properties. It can have a positive effect on vascular endothelium and improve skeletal muscle energy metabolism [15,16,17,18,19]. Given its multiactivity, the 1-MNA molecule appears to be a good candidate for treating COVID-19 survivors with chronic fatigue. Therefore, we investigated whether 1-MNA supplements could improve exercise tolerance and decrease fatigue among patients recovering from SARS-CoV-2.

## 2. Methods

### 2.1. Patients and Eligibility Criteria

Patients from registered ClinicalTrials.gov identifier—NCT04961476 were included in the analysis. All subjects from individual groups were informed about the research assumptions and gave their written consent to participate.

The decisive criteria for including patients in the study were:

1. SARS-CoV-2 virus infection was confirmed by the RT-PCR test result in accordance with the current guidelines of the Ministry of Health of Poland with home isolation (no hospitalization);
2. Patients expressing subjective feelings of limited tolerance to exercise, and above 50% greater fatigue, compared with their pre-COVID-19 levels (symptoms must have continued for at least four weeks since the last symptoms of infection);
3. No myocardial infarction, no heart failure diagnosis before COVID-19;
4. No pulmonary diseases;
5. Age ≥ 18 years;
6. Consent of the respondent to participate in the study.

Exclusion criteria:

1. Cardiological or pulmonological complications after COVID-19;
2. Anemia in blood test count;
3. Hypothyroidism and/or hyperthyroidism;
4. Renal insufficiency with eGFR < 60 mL/min.

The patients’ medical data were collected on the basis of the STOP-COVID registry survey during the patients’ direct visit to the medical center.

### 2.2. Study Protocol

The study population was composed of pts who had recovered from symptomatic COVID-19, expressing subjective feelings of limited tolerance to exercise and above 50% greater fatigue, compared with their pre-COVID-19 levels. These symptoms must have continued for at least four weeks since the last symptoms of infection. Patients were included in the study who had their last symptoms of infection between 4 weeks and 3 months prior to the start of the study. Patients with cardiological and pulmonological complications that could affect the symptoms of reduced exercise tolerance were excluded. Chronic obstructive pulmonary disease and/or asthma patients were also excluded from the study. The selected pts were randomized into two groups:

1. Gr 1: NO-1-MNA—without supplementation;
2. Gr 2: 1-MNA—with 1-MNA supplementation. 1-MNA supplements were taken once a day at a dose of 58 mg, in the morning after a meal.

The study enrolled 50 consecutive patients who met the inclusion criteria for the study. Basic statistical criteria were used to calculate the minimum sample size, taking into account the absolute standard deviation of estimate, the level of significance and the statistical power of the test. With the multitude of variables subject to analysis, it was estimated that the minimum number assumed in the study would enable the collection of reliable data that could be a starting point for further research hypotheses in order to conduct in-depth population studies. Pts were randomized according to the order in which they entered the study—one patient to the 1-MNA group, the next patient to NO-1-MNA, etc.

At the beginning of the study (Phase 0), pts from both groups were given the following examinations:

- oxygen saturation assessment performed with a pulse oximeter;
- heart rate monitoring;
- dyspnea assessment according to the Borg scale;
- assessment of fatigue with the Fatigue Severity Scale (FSS) test questionnaire.

The primary endpoint was improvement in performance as assessed by the 6MWT test, and the secondary endpoint was defined as reduction in fatigue by FSS assessment.

### 2.3. Fatigue Assessment

The FSS is a self-administered questionnaire for assessing the severity of fatigue in different situations over the past week. Each item is rated on a scale from 1 to 7, where 1 indicates strong disagreement and 7 strong agreement. The final score is the mean value of the 9 items. In accordance with previous studies, we considered values ≥4 as indicating severe fatigue [20,21,22].

In both groups, a 6-minute walk test (6MWT) was carried out according to the protocol described in the American Thoracic Society statement on the 6MWT in 2002 [23]. Patients were instructed to walk the greatest distance possible in 6 minutes, at a self-determined pace, pausing to rest if needed. Walking distance in meters, heart rate (HR), oxygen saturation and dyspnoea, rated from 1 to 10 on the Borg CR-10 dyspnoea scale, were assessed before and after the test.

After 1 month (Phase 1), a follow-up FSS and 6MWT were performed in both groups. The following parameters were assessed and compared with the results in Phase 0:

- distance, and the mean difference in distance, in 6MWT;
- number and percentage of pts with improved distance in 6MWT;
- FSS score;
- number and percentage of patients with FSS ≥ 4.

### 2.4. Bioethics Approval

The study was carried out in conformance with the Declaration of Helsinki, and approval from the Bioethics Committee of Lodz Regional Medical Chamber to conduct the study was obtained—no. 0115/2021. All subjects from individual groups were informed about the research assumptions and gave their consent to participate in it.

### 2.5. Statistical Analysis

The data was coded and entered into Microsoft Office Excel and STATISTICA version 13.1. The most important computational methods in the field of descriptive statistics were used in the statistical analysis, including measures of the distribution of measurable features: the arithmetic mean to calculate the average level of the analyzed statistical feature in the population; the standard deviation for the assessment of the dispersion of measurable features; the median to calculate the median value of the feature for the study group when the distribution of the feature deviated from the normal distribution. The structure indexes interpreted in fractions were also calculated (for <100), to assess the ratio of a part of a given statistical population distinguished by a specific level/variant of a feature to the entire studied population. On the basis of these methods, the relationships between the selected statistical features were calculated and the significance of differences in the selected variables was assessed. The χ^2^ test of independence was used to test the relationship between nominal variables. The Student’s *t*-test, the U–Mann–Whitney test, the Wilcoxon pairs test and the difference test were used to compare the two groups in terms of the quantitative variables. The Shapiro-Wilk test was used to assess the normality of the distribution. The research hypotheses were verified on the basis of a significance level *p* ≤ 0.05.

## 3. Results

In total, 50 pts were included in the study: 16 males, 34 females. The groups were randomized: Gr NO-1-MNA—25 pts, without supplementation; Gr 1-MNA—25 pts with MNA-1 supplementation. The clinical characteristics of the groups in Phase 0 are presented in Table 1.

In each group, there were 4 pts with comorbidities: in Gr 1-MNA, 4 pts had arterial hypertension; in Gr NO-1-MNA, 3 patients had arterial hypertension and one patient had Type 2 diabetes mellitus. None of the patients had clinical symptoms of heart failure and echocardiography showed no abnormalities in the structure or function of the pts’ hearts. The pts’ medications were not changed during the supplementation period. All patients had correct ambulatory blood pressure. There were no significant differences between the two groups in terms of the mean age, BMI, oxygen saturations, distance in 6MWT, dyspnea on the Borg scale, or fatigue assessment on the FSS scale in Phase 0.

### 3.1. Results after One Month with MNA-1 Supplementation

#### 3.1.1. Six-Minute Walk Test

**Figure 1:**
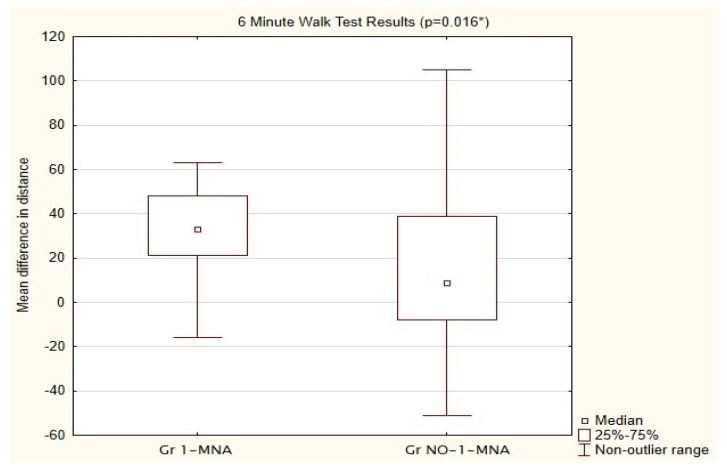
Comparison of mean differences in distance between Group NO-1-MNA and 1-MNA in 6MWT after 1 month supplementation with 1-MNA. Gr NO-1-MNA—patients without supplementation; Gr 1-MNA-patients with 1-MNA supplementation; 6MWT—6-min walk test. * U—Mann−Whitney test.

A significant improvement in the mean distance covered in the 6MWT was noted among the pts in Gr 1-MNA, compared with those in Gr NO-1-MNA. Patients with MNA doubled the distance difference after 1 month (Phase 1) compared with Gr NO-1-MNA (mean Gr NO-1-MNA was 18.14 m vs. 38.86 m in Gr 1-MNA). The results are shown in Figure 1.

**Figure 2:**
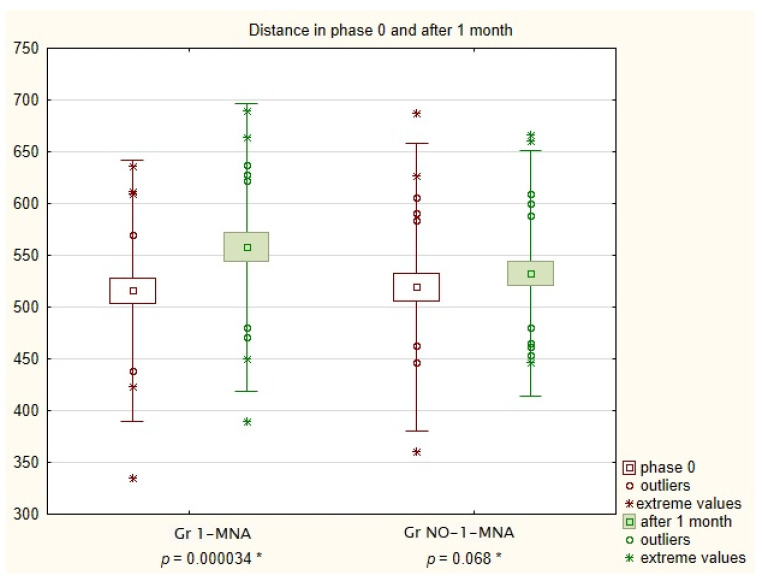
Mean distance for Groups NO-1-MNA and 1-MNA in 6MWT after 1 month of supplementation with 1-MNA. Gr NO-1-MNA—patients without supplementation; Gr 1-MNA—patients with 1-MNA supplementation; 6MWT—6-min walk test. * the Wilcoxon pairs test.

We also noted that in Gr 1-MNA, the 6MWT distance was significantly higher after 1 month of supplementation with 1-MNA, compared with the beginning of the study (515.18 m in Phase 0 vs. 557.8m in Phase 1; *p* = 0.000034). In Gr NO-1-MNA, the distance also increased, but the difference was not statistically significant (519.24 m in Phase 0 vs. 532.52 m in Phase 1; *p* = 0.068). The results are shown in Figure 2.

In Gr 1-MNA, significantly more pts improved their distance in the 6MWT (23 out of 25 pts, equal to 92%), by a mean of 47 m, compared with Gr NO-1-MNA (15 of 25 pts, equal to 60%; *p* = 0.0061). Only 2 pts in Gr 1-MNA did not improve their distance. However, these 2 pts reported less fatigue in the FSS. The observed changes in distance in the 6 min walk test in the groups Gr NO-1-MNA and Gr 1-MNA at Phase 0 and after 1 month are shown in Table 2.

#### 3.1.2. Fatigue

Interestingly, after one month, both groups reported an increase in fatigue in the FSS (Gr NO-1-MNA FSS = 4.53 in Phase 0 vs. FSS = 4.94 in Phase 1, and Gr 1-MNA FSS = 4.23 in Phase 0 vs. FSS = 4.42 in Phase 1). As in the comparison of FSS in Phase 1, the differences between Gr NO-1-MNA and Gr 1-MNA were not statistically significant.

In Phase 0, five pts (20%) in Gr 1-MNA had an FSS score ≥ 4. In Gr NO-1-MNA the number of patients with an FSS score ≥ 4 was six (24%) (NS). After one month, significantly more patients in the group without 1-MNA had severe fatigue (FSS ≥ 4) compared with the group with supplementation (Gr 1-MNA = 5 pts (20%) vs. Gr NO-1-MNA = 14pts (56%); *p* = 0.008).

## 4. Discussion

To the best knowledge of the authors, this is the first study to assess the effect of supplements on the performance parameters of patients after COVID-19. Our results show a significant improvement in walking distance during the 6MWT test following supplementation with 1-MNA. Both the distance and the mean difference significantly increased after 1 month in the group with 1-MNA supplementation. As many as 95% of the pts with 1-MNA supplementation showed an increase in walking distance. Moreover, significantly fewer patients reported significant fatigue, defined as an FSS score ≥ 4, after one month of supplementation, compared with pts without 1-MNA supplementation.

To date, there is still no specific treatment for patients with post-COVID-19 syndrome. The bulk of research work has rightly focused on prevention and treatment of the acute phase of the disease [14]. Only rehabilitation has been considered as a treatment for improving respiratory function, exercise tolerance, fatigue and quality of life in pts after COVID-19 [24,25]. Fatigue and limited tolerance to exercise are the leading symptoms of Long COVID in hospitalized and not-hospitalized sequelae [1,26,27,28]. Fatigue and limited exercise tolerance following viral infection have a very complex pathology. Effective treatment requires supplementation with a substance that has comprehensive action: protecting the vascular endothelium, as well as having anti-inflammatory and anti-thrombotic effects [10,13].

Many studies show that 1-MNA possesses anti-thrombotic and anti-inflammatory activity and also increases the lifespan of *Caenorhabditis elegans* through SIRT1-dependent mitohormetic effects [15,16,17]. 1-MNA has been found to increase the production of prostacyclin (PGI2) in endothelial cells in rodent models of thrombosis [15]. 1-MNA also increases the bioavailability of NO in the vascular endothelium and regulates the impaired activity of endothelial nitric oxide synthase [18,29]. The endogenous level of 1-MNA rises during physical exercise, and 1-MNA is regarded as a signaling molecule produced in skeletal muscle coordinating energy metabolism [19]. Fatigue, often caused by acute viral infections such as influenza and COVID-19, may be associated with an insufficient endogenous level of 1-MNA [30]. Przyborowski et al. [31] studied the effects of 1-MNA on exercise capacity and the endothelial response to exercise in diabetic mice. Eight-week-old mice were given 1-MNA for 4 weeks, and their exercise capacity and endurance running were assessed. The 1-MNA-treated mice showed significantly more resistance to fatigue in endurance exercises than the control (mice without supplementation). Schmeisser et al. [17] also noted the beneficial effect of 1-MNA, demonstrating that this compound increased the mean velocity of nematode movement. It has also been shown that in mice muscle, activity induces 1-MNA formation, confirming its role in exercise [32]. In humans, methylation of NA metabolites by NNMT as well as 1-MNA supplementation may become versatile approaches to extend health span [33].

In an in vivo study, Chlopicki et al. [15] found that pharmacological doses of 1-MNA acutely increase secretion of prostacyclin (PGI2) from endothelial cells and may regulate thrombotic as well as inflammatory processes in rodent models of thrombosis. Another positive effect of 1-MNA for endothelium is to enhance the release of nitric oxide from endothelial cells [18]. Ström suggests that 1-MNA could have a beneficial effect on muscle function, by enhancing the utilization of energy stores in response to low muscle energy availability [19]. Others have proposed that activating SIRT1 has a positive effect on muscle function. The literature thus provides very significant evidence that 1-MNA acts in a complex way, which could make it a valuable clinical adjunct to the treatment of fatigue and exercise limitation after COVID-19. Given the available evidence, the use of 1-MNA to prevent inflammation damage associated with COVID-19 seems rational.

The 6MWT has long been used in cardiac and pulmonary rehabilitation as an objective assessment of the physical capacity of patients and the effects of treatment, including training programs [34,35]. The test has also been used to study pts after contracting COVID-19 [36,37]. Numerous studies have shown improvements in the 6MWT walking distance among post-hospital patients [38,39,40]. However, there have been no published studies assessing the results for 6MWT in pts without hospitalization.

In our study, which included non-hospitalized pts, the mean distance in the 6MWT of more than 500 m was much higher than the distances reported for hospitalized patients (333 m in Ahmed M Abodonya AM et al. [41], or 240 m in Curci et al. [42]). Of course, this discrepancy may be due to the milder course of COVID-19. In Wonga et al.’s [43] study on pts with mild COVID-19, the mean distance (491 m) was similar to that in our study.

The difference in the average distance we obtained with 1-MNA supplementation was comparable to the results for pulmonary rehabilitation (44 m) after COVID-19 reported by Abodonya et al. [41]. In a study by Spielmanns et al. [44], a group of patients after COVID-19 were given 30 treatment sessions over 3 weeks of comprehensive pulmonary rehabilitation. The distance difference in the 6MWT was on average 180 (±101) meters for the rehabilitated group. This difference was many times greater than what we obtained during 1-MNA supplementation. Similarly, in a study by Tazato et al. [45], the improvement in distance was much greater than in our 1-MNA group, but the follow-up time was longer (3 months).

As in the above-mentioned studies, almost all the pts in our study in Gr 1-MNA improved their performance in the 6MWT.

Fatigue and significant reductions in tolerance to exercise are among the most common persistent symptoms after COVID-19 [25,46,47]. Fatigue is a common consequence of several viral infections, as shown in the case of EBV by White et al. [48]. It is still unclear why chronic fatigue and the other long-term complications persist in some COVID-19 patients. However, most researchers and clinicians agree that long-term COVID-19 symptoms are associated with the ability of coronavirus to trigger a massive inflammatory response [49].

The FSS is an established research tool [20]. The average score for FSS among the patients in our study was much higher than in the healthy population and comparable with pts with multiple sclerosis (MS) or after a stroke. Fatigue after COVID-19 is thus a significant clinical problem. 1-MNA supplementation did not result in a reduction in the number of people reporting fatigue. However, the low percentage of 1-MNA-treated pts with significant fatigue defined as FSS ≥ 4 was comparable to that in the healthy population (18% in [20] and 20% in our study). This is a very promising result after such a short period of time. There were statistically significantly fewer patients with severe fatigue in the 1-MNA group. On the other hand, the percentage of pts in the control group (without 1-MNA supplementation) with severe fatigue was similar to that among pts with MS or after a stroke. The anti-inflammatory effect of 1-MNA, which appears to have an impact on fatigue after SARS-CoV-2, may have had a decisive influence on this result.

Currently, there is no evidence that nutrition or physical exercise can contribute to the recovery of patients with fatigue [14]. A clinical trial is ongoing into the use of supplemental creatine to treat chronic fatigue syndrome, but no results have been published yet [8]. The main limitation of the present study was the short observation time. It would be interesting to see if this initial effect would be sustained over a longer follow-up period. It cannot be ruled out that the 1-MNA group experienced a placebo effect. A randomized 1-MNA vs. placebo study on a larger group of patients over a longer observation period is, therefore, also needed. However, our preliminary results suggest that supplementation with 1-MNA is a promising treatment for Long COVID in patients without heart or lung disease and without cardiopulmonary complications.

## 5. Conclusions

Based on the results obtained, we have drawn the following conclusions:

1. 1-MNA supplementation significantly improved physical performance in a 6-minute walk test and reduced the percentage of patients with severe fatigue after COVID-19;
2. The comprehensive action of 1-MNA, including anti-inflammatory and anticoagulant effects, as well as activation of the SIRT1 enzyme, may be beneficial for the recovery of patients with persistent symptoms of fatigue and low tolerance to exercise after COVID-19.

## Figures and Tables

**Table 1:** Clinical characteristics of the groups Gr 1-MNA and Gr NO-1-MNA at Phase 0.

Parameters		Gr 1-MNA *n* = 25 M ± SD 95% CI	Gr NO-1-MNA *n* = 25 M ± SD 95% CI	*p*-Value
**Age**		47.40 ± 9.73 43.38–51.42	49.60 ± 10.55 45.24–53.96	0.447
**Median**		**49**	50	
**Sex**	**Female**	17 (0.68)	17 (0.68)	1.000
	**Male**	8 (0.32)	8 (0.32)	
**BMI (kg/m** **^2^** **)**		26.88 ± 4.69 24.94–28.82	27.17 ± 4.92 25.14–29.20	0.834
**Median**		**25.97**	26.42	
**Heart rate at rest in Phase 0**		72.40 ± 11.74 67.55–77.25	78.64 ± 1.32 78.10–79.18	0.352
**Median**		**76**	79	
**Oxygen saturation at rest in Phase 0**		97.28 ± 2.03 96.44–98.12	96.96 ± 4.71 95.02–98.90	0.522
**Median**		**98**	98	
**Distance in 6 min walk test in Phase 0**		515.8 ± 63 512.24–519.36	519.2 ± 69 490.72–547.68	0.850
**Median**		**516**	504	
**Fatigue assessment according to Fatigue Severity Score in Phase 0**		4.28 ± 1.38 3.71–4.85	4.53 ± 1.16 4.05–5.01	0.490
**Median**		**4.44**	4.54	
**Dyspnoea on the Borg Scale at rest in Phase 0**				**0.128**
**0**		**8 (0.32)**	7 (0.28)	
**1**		**3 (0.12)**	2 (0.08)	
**2**		**9 (0.36)**	3 (0.12)	
**3**		**2 (0.08)**	9 (0.36)	
**4**		**2 (0.08)**	2 (0.08)	
**5**		**1 (0.04)**	2 (0.08)	

M—mean; SD—standard deviation; 95%CI—95% confidence interval; BMI—body mass index, derived from the mass (weight) and height of a person. The BMI is defined as the body mass divided by the square of the body height, and is expressed in units of kg/m^2^, resulting from mass in kilograms and height in meters. Gr NO-1-MNA—patients without supplementation; Gr 1-MNA—patients with MNA-1 supplementation; *p*—statistical significance. Bolds means highlight data.

**Table 2:** Distance in 6 min walk test in the groups Gr NO-1-MNA and Gr 1-MNA at Phase 0 and after 1 month.

Parameters	Gr 1-MNA *n* = 25	Gr NO-1-MNA *n* = 25	Absolute Mean Difference Between Groups	*p*-Value
Mean distance in phase 0	515.80	519.24	3.44	0.000034
Mean distance after 1 month	557.80	532.52	25.28	0.068
Mean difference between distances	42.00	13.28	28.72	0.0016

Gr NO-1-MNA—patients without supplementation; Gr 1-MNA—patients with MNA-1 supplementation; *p*—statistical significance.

## Data Availability

The data underlying this article cannot be shared publicly, to protect the privacy of individuals that participated in the study.
